# Supplementary material for: Identification of Beneficial Microbial Consortia and Bioactive Compounds with Potential as Plant Biostimulants for a Sustainable Agriculture
Source: Microorganisms. 2021 Feb 19;9(2):426. doi: 10.3390/microorganisms9020426 (PMC7922931; doi:10.3390/microorganisms9020426)
Supplement: Supplementary file 1 [file microorganisms-09-00426-s001.zip › Supplementary files /microorganisms-1081774_Supplementary.pdf]

microorganisms

MDPI

Supplementary

Identification of Beneficial Microbial Consortia and Bioactive Compounds with Potential as Plant Biostimulants for a Sustainable Agriculture

Silvia Tabacchioni [1,†], Stefania Passato [2], Patrizia Ambrosino [2], Liren Huang [3], Marina Caldara [4], Cristina Cantale [1,†], Jonas Hett [5], Antonella Del Fiore [1], Alessia Fiore [1], Andreas Schlüter [3], Alexander Sczyrba [3], Elena Maestri [4], Nelson Marmiroli[4], Daniel Neuhoff [5], Joseph Nesme [6], Søren Johannes Sørensen [6], Giuseppe Aprea [1], Chiara Nobili [1], Ombretta Presenti [1], Giusto Giovannetti [7], Caterina Giovannetti [7], Anne Pihlanto [8], Andrea Brunori [1] and Annamaria Bevivino [1,*]

[1] Department of Sustainability, Italian National Agency for New Technologies, Energy and Sustainable Economic Development, ENEA Casaccia Research Center, 00123 Rome, Italy; silvia.tabacchioni@enea.it (S.T.); cristina.cantale@enea.it (C.C.); antonella.delfiore@enea.it (A.D.F.); alessia.fiore@enea.it (A.F.); giuseppe.aprea@enea.it (G.A.); chiara.nobili@enea.it (C.N.); ombretta.presenti@enea.it (O.P.); andrea.brunori@enea.it (A.B.); annamaria.bevivino@enea.it (A.B.)

[2] AGRIGES srl, 82035 San Salvatore Telesino (BN), Italy; stefania.passato@agriges.com (S.P.); patrizia.ambrosino@agriges.com (P.A.)

[3] Center for Biotechnology (CeBiTec), Institute of Crop Science and Resource Conservation, Bielefeld University, 33615 Bielefeld, Germany; huanglr@cebitec.uni-bielefeld.de (L.H.); aschluet@cebitec.uni-bielefeld.de (A.S.); asczyrba@cebitec.uni-bielefeld.de (A.S.)

[4] SITEIA.PARMA, Interdepartmental Centre for Food Safety, Technologies and Innovation for Agri-food and Department of Chemistry, Life Sciences and Environmental Sustainability, 43124 University of Parma, Italy; marina.caldara@unipr.it (M.C.); elena.maestri@unipr.it (E.M.); nelson.marmiroli@unipr.it (N.M.)

[5] Department of Agroecology & Organic Farming Rheinische Friedrich-Wilhelms-Universität Bonn, 53121 Bonn, Germany; jhett@uni-bonn.de (J.H.); d.neuhoff@uni-bonn.de (D.N.)

[6] Department of Biology, University of Copenhagen, Universitetsparken 15 Bldg 1, 2200 Copenhagen, Denmark; joseph.nesme@bio.ku.dk (J.N.); sjs@bio.ku.dk (S.J.S.)

[7] Centro Colture Sperimentali, CCS-Aosta S.r.l., 11020 Quart (AO), Italy; giustogiovannetti@hotmail.com (G.G.); caterina@micosat.it (C.G.)

[8] Natural Resources Institute Finland (Luke), Myllytie 1, 31600 Jokioinen, Helsinki, Finland; anne.pihlanto@luke.fi (A.P.)

* Correspondence: annamaria.bevivino@enea.it

† These authors contributed equally to this work.

Citation: Tabacchioni, S.; Passato, S.; Ambrosino, P.; Huang, L.; Caldara, M.; Cantale, C.; Hett, J.; Del Fiore, A.; Fiore, A.; Schlüter, A.; et al. Identification of Beneficial Microbial Consortia and Bioactive Compounds with Potential as Plant Biostimulants for a Sustainable Agriculture. *Microorganisms* **2021**, *9*, x. https://doi.org/10.3390/xxxxx

Academic Editor: Denis Faure

Received: 01 January 2021

Accepted: 08 February 2021

Published: date

Publisher's Note: MDPI stays neutral with regard to jurisdictional claims in published maps and institutional affiliations.

Copyright: © 2021 by the authors. Submitted for possible open access publication under the terms and conditions of the Creative Commons Attribution (CC BY) license (http://creativecommons.org/licenses/by/4.0/).

Figure S1. Genome coverage comparison after peak removal. A), Reads coverage of the *Burkholderia ambifaria* MC40-6 strain genome sequence is presented by the mean coverage of a 1000-nucleotides window. Red arrows point out highly covered peak regions of 16S genes. After peak removal process, the highly covered regions are no longer present in B). Both A) and B) are plotted using the fragment recruitment results of the same *B. ambifaria* MC40-6 strain and the sequencing data from NCBI with accession ID: SRR5487771

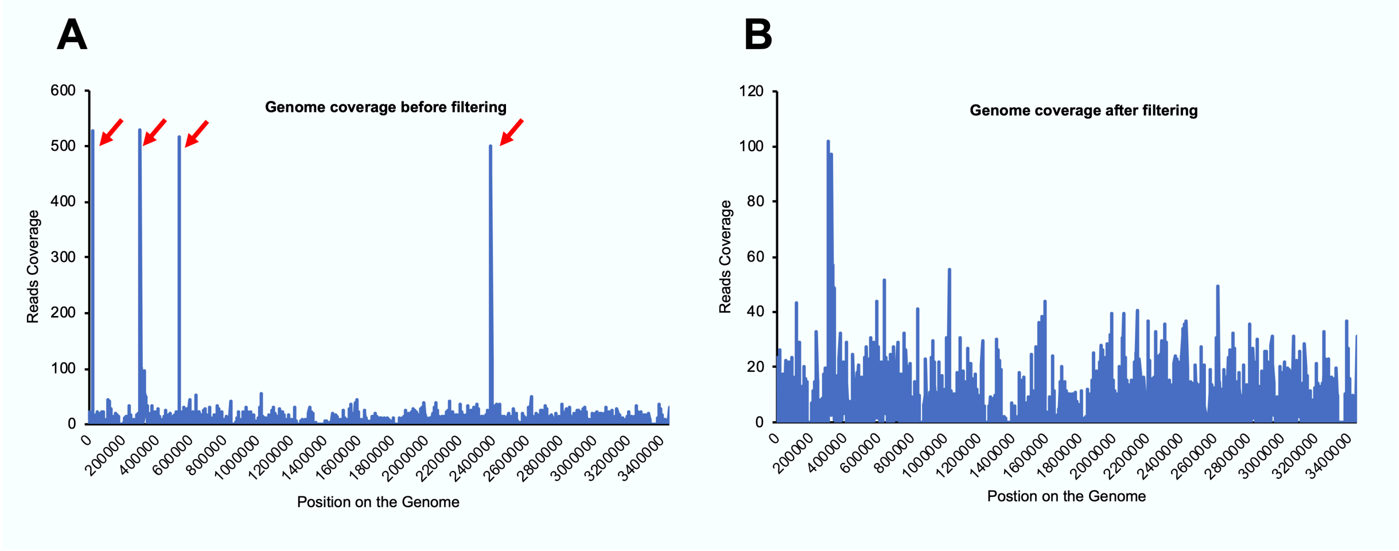

Data Sheet S1. Data collected from articles retrieved via systematic literature survey. To proceed with the preliminary screening of publications, privileging those presenting more consistent, significant and representative data, scientific articles received an individual score made up by the sum of marks assigned for each of the following six indicators, with possible alternatives and relative marks shown in parentheses: i) Experimental environment (growth-chamber=0, greenhouse=1 field=2); ii) PGPMs ease and readiness of acquisition (patent=0, research institute=1,commercial= 2); iii) Experimental design-number of treatment combinations (up to 5=1, 6 to 10=2, 11 to 15=3, over 15=4); iv) Experimental design-number of measured parameters (up to 5=1, 6 to 10=2, 11 to 15=3, over 15=4); v) PGPMs characterisation at molecular level (no=0, yes=1); vi) Overall evaluation of PGPMs effectiveness (low=0, moderate=3, high=6, plus one additional extra point in all cases if the experiment was carried out without the employment of chemical inputs). Rows highlighted in grey and in white refer respectively to articles whose total mark resulted below or above the discriminative threshold adopted, set at 10 points. Only the latter group of publications (73 out of 134) was considered in the subsequent steps of PGPMs selection.

Table S1. The genomes of the selected PGP strains isolated from rhizosphere.

Table S2. The genomes of the selected PGPM isolated from soil samples.

Table S3. The genomes of the selected PGPM isolated from root samples.

Table S4. Database summary as related to the number of eligible articles, studies, PGPMs and consortia examined in the present study.

Crop	Eligible articles[a]	Field studies	Studies without stresses	Studies on abiotic stress	Studies on biotic stress	PGPMs	Consortia

TOMATO	41 (12)	15	15	6 - salt<br>1 - salt+drought<br>2 - drought<br>1 - cold<br>1 - flooding	1 - *Botrytis cinerea*<br>6 - *Fusarium oxysporum* f. sp. *lycopersici*<br>1 - *Fusarium solani*<br>1 - *Helicoverpa armigera*<br>4 - *Meloidogyne incognita*<br>1 - *Phytophthora infestans*<br>3 - *Ralstonia solanacearum*<br>2 - *Rhizoctonia solani*<br>1 - *Sclerotinia minor*<br>1 - *Sclerotinia sclerotiorium*<br>1 - *Verticillium dahliae*	41	11
MAIZE	43 (31)	22	28	5 - salt<br>1 - salt+drought<br>7 - drought	1 - *Fusarium oxysporum*<br>1 - *Fusarium moliniforme*	48	11
POTATO	16 (9)	9	9	1 - drought<br>1 - drought+salt+ heavy metals<br>1- heat	1 - *Ralstonia solanacearum*<br>2 - *Rhizoctonia solani*<br>1 - *Streptomyces bottopensis*<br>1 - *Streptomyces scabies*	24	2
WHEAT	34 (21)	10	20	9 - salt<br>3 - drought<br>1 - heavy metals<br>1 - temperature	1 - *Aspergillus niger*<br>3 - *Fusarium graminearum*<br>3 - *Gaeumannomyces graminis var. tritici*<br>1 - *Heterodera avenae*<br>1 - *Macrophomina phaseolina*<br>1 - *Microsporum gypseum*<br>1 - *Penicillium chrysogenum*<br>2 - *Pythium* sp.<br>2 - *Pythium ultimum*<br>1 - *Rhizoctonia solani*	61	16

[a] In parentheses, the number of articles that reached the minimum rank value (>10).

Table S5. PGPMs and commercial biofertilizers derived from the eligible studies on tomato, maize, potato and wheat.

CROP	PGPMs	COMMERCIAL BIOFERTILIZER (Country-Company/Species/Effect)
TOMATO	*Achromobacter piechaudii, Alcaligenes* sp., Arbuscular mycorrhizal fungi (AMF), *Azotobacter chroococcum, Bacillus* sp., *Bacillus amyloliquefacien, Bacillus cereus, Bacillus chitinosporns, Bacillus laterosporus, Bacillus licheniformis, Bacillus megaterium, Bacillus methylotrophicus, Bacillus pumilus, Bacillus simplex, Bacillus subtilis, Beauveria bassiana, Chryseobacterium* sp., *Citrobacter youngae,*	BioF (Bangladesh-Natore Develop Soc/*Trichoderma hartianum* T22/PGP)<br>BioRex 1 (Hungary-BioRex/*Bacillus subtilis*+ *Bacillus thuringiensis*+*Bacillus megaterium*/PGP)<br>BioRex 2 (Hungary-BioRex/*Azotobacter chroococcum*+*Azospirillum lipoferum*+*Pseudomonas putida*/PGP)<br>Biostart® 2000 (South Africa-Microbial Solutions ltd/

	Combifector B formulation (*Trichoderma harzianum* OMG16 and *Bacillus amyloliquefaciens* FZB42, enriched with zinc and manganese), *Enterobacter* sp., *Enterobacter cloacae, Flavobacterium glaciei, Klebsiella* sp., *Klebsiella pneumoniae, Lactobacillus paracasei, Paenibacillus polymyxa, Pleosporales* (suborder *Massarineae*), *Pseudomonas* sp., *Pseudomonas aeruginosa, Pseudomonas brassicacearum, Pseudomonas chlororaphis, Pseudomonas frederiksbergensis, Pseudomonas fluorescens, Pseudomonas migulae, Pseudomonas protegens, Pseudomonas putida, Pseudomonas stutzeri, Pseudomonas vancouverensis, Rhizophagus intraradices, Serratia marcescens, Streptomyces* sp., *Streptomyces rubrogriseus, Trichoderma* sp., *Trichoderma harzianum*	*Bacillus chitinosporns+Bacillus laterosporus+ Bacillus licheniformis*/PGP)<br>NemOut® (Brazil/*Bacillus licheniformis+ Bacillus subtilis+Trichoderma longibrachiatum*/ biocontrol)<br>Paecilomyces JCO® (Brazil/*Purpureocillium lilacinus*/biocontrol)<br>Rizotec (Brazil/*Poconia chlamydosporia*/biocontrol)<br>Serenade® SC (Germany-Bayer/*Bacillus subtilis*/ biocontrol)<br>Nemat® WP (Brazil/*Purpureocillium lilacinus*/biocontrol)<br>Pochar™ (Italy/Microspore/*Arthrobotis oligospora +Glomus sp+Bacillus spp.*/biocontrol)<br>Polygardon (Slovakia-Polyversum/*Phythium oligandrum*/biocontrol)<br>Phylazonit MC® (Hungary/*Pseudomonas putida+ Azotobacter chroococcum+Bacillus circulans+Bacillus megaterium*/PGP under water stress);<br>FZB24®WG (Germany-ABiTEP/*Bacillus amyloliquefaciens* FZB24/PGP)<br>Rhizovital FZB42® (Germany-ABiTEP/*Bacillus amyloliquefaciens* FZB42/PGP)
MAIZE	*Acinetobacter* sp., *Achromobacter* sp., *Azospirillum* sp., *Azospirillum brasilense, Azospirillum lipoferum, Azotobacter* sp., *Azotobacter chroococcum, Bacillus* sp., *Bacillus amyloliquefaciens, Bacillus cereus, Bacillus lentus, Bacillus megaterium, Bacillus pumilus, Bacillus subtilis, Burkholderia* sp., *Burkholderia ambifaria, Burkholderia phytofirmans, Chryseobacterium humi, Chryseobacterium palustre, Glomus intraradices, Enterobacter* sp., *Enterobacter aerogenes, Enterobacter radicincitans, Meyerozyma guilliermondii, Mitsuaria* sp., *Mycobacterium phlei, Paenibacillus polymyxa, Proteus* sp., *Pseudomonas* sp., *Pseudomonas aeruginosa, Pseudomonas alcaligenes, Pseudomonas aurantiaca, Pseudomonas entomophila, Pseudomonas fluorescens, Pseudomonas monteilli, Pseudomonas*	Trianum® (Netherlands- Koppert B.V./*Trichoderma harzianumT*-22/PGP)<br>Proradix® (Germany- Sourcon-Padena GmbH /*Pseudomonas* sp.DSMZ 13134/ PGP)<br>Rhizovital FZB42® (Germany-ABiTEP gmbH/*Bacillus amyloliquefaciens*/PGPR)<br>AM fungal inoculum (Italy-Mybasol srl/*Rhizophagus intraradices+Glomus aggregatum+Glomus viscosum+ Claroideoglomus etunicatum+Claroideoglomus claroideum*/PGP)<br>Rhizoflo Premium Maìz® (Argentina/*Azospirillum brasilense + Pseudomonas fluorescens*/PGP)<br>*Azospirillum lypoferum* CRT1 (France-Lesaffre Agrauxine/*Azospirillum lypoferum* CRT1/PGP)

	putida, Pseudomonas stutzeri, Pseudomonas syringae, Pseudomonas thivervalensis, Ralstonia sp., *Serratia* sp., *Serratia mercescens, Serratia proteamaculans, Sphingobacterium* sp., *Sphingomonas* sp., *Trichoderma atroviride, Trichoderma harzianum, Trichoderma virens*	
POTATO	*Achromobacter xylosoxidans, Actinobacter* sp., *Agrobacterium* sp., *Azotobacter* sp., *Azospirillum* sp., *Bacillus* sp., *Bacillus cereus, Bacillus firmus, Bacillus pumilus, Bacillus subtilis, Brevibacillus laterosporus, Enterobacter* sp., *Methylobacterium* sp., *Paenibacillus dendritiformis, Paenibacillus macerans, Pseudomonas* sp., *Pseudomonas fluorescens, Pseudomonas koreensis, Pseudomonas oryzihabitans, Rhizobium* sp., *Rhizophagus irregolaris, Serratia marcescens,Tricoderma harzianum, Variovorax paradoxus*	Pochar™ (Italy-Microspore/*Pochonia chlamydosporia+Arthrobotis oligospora+Glomus* sp.+*Bacillus* spp./biocontrol)<br>EM (China/effective microorganism mixture/PGP)<br>KLEPS-Ko (Ukraine/*Paenibacillus* sp., *Klebsiella oxytoca, Pantoea agglomerans*, three *Pseudomonas* strains and *Stenotrophomonas maltophilia*/PGP)
WHEAT	*Aeromonas* sp., *Alcaligenes* sp., *Arthrobacter* sp., *Azospirillum brasilense, Azotobacter chroococcum, Azotobacter vinelandii, Brevibacterium halotolerans, Bacillus* sp., *Bacillus cereus, Bacillus subtilis, Bacillus insolitus,Bacillus pumilus, Bacillus megaterium, Bacillus safensis, Bacillus thuringiensis, Brevundimonas* sp., *Microbispora* sp., *Micromonospora* sp., *Micromonospora aurantiaca, Mycobacterium* sp., *Mycobacterium phlei, Mycoplana bullata, Nocardioides albus, Nocardioides lucentensis, Ochrobactrum pseudogregnonense,Pantoea* sp., *Pantoea agglomerans, Paenibacillus* sp., *Paenibacillus polymyxa, Penicillium bilaii, Penicillium roqueforti, Piriformospora indica, Providencia* sp., *Pseudomonas aurantiaca, Pseudomonas aeruginosa, Pseudomonas chlororaphis, Pseudomonas extremorientalis, Pseudomonas fluorescens, Pseudomonas putida, Rhodococcus rhodochrous, Serratia marcescens, Stenotrophomonas maltophilia, Streptomices* sp., *Streptomices carpinensis, Streptomices cellulosae, Streptomices cheonanensis, Streptomices coelicolor, Streptomyces geysiriensis, Streptomyces griseus, Streptomyces lomondensis,*	Macrogen (South Korea/*Brevibacterium halotolerans* FAB3/PGP)<br>RIZOFLO Liquid (Argentina/*Azospirillum brasilense* Az1/ PGP)<br>NoctinAZO (Argentina/*Azospirillum brasilense* Az2/PGP)<br>RIZOFOSliq (Argentina/*Pseudomonas fluorescens* Pf/PGP)<br>Trianum-P (Netherlands/*Trichoderma harzianum* T22/PGP)<br>Proradix (Germany/*Pseudomonas* sp. DSMZ 13134/PGP)<br>RhizoVital 42 (Germany/*Bacillus amyloliquefaciens ssp. plantarum* FZB42/PGP)<br>Bio-DC (Germany /*Penicillium* sp./PGP)<br>BactoProf (Germany/*Trichoderma harzianum and five species of Bacillus*/PGP)<br>Trichodermin B (Islamic Republic of Iran-Talphigh Daneh Company/ *Trichoderma harzianum*/biocontrol)<br>Subtilin (Islamic Republic of Iran-Talphigh Daneh Company/ *Bacillus subtilis*/biocontrol)

	Streptomyces olivaceus, Streptomyces rochei, Streptomyces thermolilacinus, Streptomyces tricolor, Streptomyces werraensis, Trichoderma brevicompactum, Trichoderma longibrachiatum, Trichoderma koningii, Trichoderma koningiopsis, Trichoderma virens, Trichoderma viridescens

Table S6. Carriers and delivery methods for agricultural application of PGPM.

Carrier and/or delivery methods	Recorded applications	Advantages	Disadvantages	Selected references
Alginate encapsulation, alone or with perlite, carrageenan or other polymers	*Azotobacter*<br>*Pseudomonas*<br>*Rhizobia*<br>Fungi	High stability along time<br>Slow release of bacteria<br>Protection from stress	Expensive<br>The loss of the compound during preparation<br>Requires additional compounds	[1]–[3]
Char or coal[a]	*Pseudomonas* (for remediation)<br>*Azospirillum*<br>*Bacillus*	Immobilizes bacteria<br>Environmentally friendly<br>Supports biofilm growth<br>Improves soil properties<br>Not degradable	Not standardised,<br>Needs characterisation<br>Possible release of phytotoxic compounds<br>Requires evaluation of pore sizes	[2],[4]–[9]
Chitosan	Mainly delivery of chemicals<br>*Pseudomonas*<br>*Azospirillum*	Elicits resistance against pathogens<br>Suitable for spraying<br>Suitable for encapsulation, with starch	Antimicrobial activity	[10]–[12]
Compost, vermicompost<br>Recent, nano-compost	Many different microbial species	Traditional<br>Familiarity<br>Provides additional benefits	Might interfere with nutrient balance<br>Unstable in time	[13],[14]
Inert materials: clay, zeolite, bentonite, montmorillonite	*Azotobacter*	Inexpensive	None reported	[1],[2]
Nanoparticles, nanotubes	Mainly encapsulation of chemicals<br>Nano-Ag Answer®	Precision delivery system	Potential for toxicity to humans<br>Difficulty in preparation	[15],[16]
Peat moss	*Azotobacter*<br>*Rhizobia* (common method)<br>*Mycorrhizae*	Stability along time<br>Easiness of preparation<br>Familiarity<br>Water-holding capacity	Scarce availability<br>Not standardised<br>High environmental impact<br>Difficult to sterilise<br>Low pH	[1],[2],[17],[18]
Perlite[b]	*Rhizobium*<br>*Bacillus*	Stability along time	None reported	[17]

Talc	*Pseudomonas, Rhizobia Trichoderma*	Inert<br>Available<br>Suitable for spraying	None reported	[2],[18]
Vermiculite[c]	*Azotobacter Pseudomonas*	Can be sterilised easily<br>Provides niches<br>Available<br>Non expensive	Large volume for packing	[1],[2],[18]

[a] Biochar has been successfully employed to deliver PGPMs, being used as a carrier material for microbial inoculants, applied as seed-coatings. Biochar is used as soil amendment as it improves several soil properties, and thanks to its porous structure, it offers a protective environment for the delivery of microbes of interest. It is also allowed in the EU as amendment in organic agriculture and can therefore be applied widely in field conditions [4],[19]–[25].

[b] Perlite is a volcanic rock composed of a little-hydrated aluminium silicate, easy to sterilize without producing toxic compounds and has been employed as a carrier [2],[18].

[c] Vermiculite is a naturally occurring layer silicate mineral easy to sterilize, widely available and not expensive. It maintains a good moisture, has a pH approximately neutral and good buffering capacities, it has plant growth promotion capacity and has been successfully used to deliver microbes to the rhizosphere [17],[26].

Table S7. Radial growth of *T. harzianum* TH01 growth at 24 h, 48 h, and 72 h cultivated on water agar (WA) plates supplemented with increasing concentrations of bioactive compounds BS1, BS2 and BS3. As control, *T. harzianum* TH01 was grown on potato dextrose agar (PDA) and WA plates.

Bioactive compounds	Treatments	24 h	48 h	72 h
**BS1**	WA	1.57 ± 0.06 **gh**	4.67 ± 0.31 **de**	6.97 ± 0.23 **c**
	WA + BS 10 ppm	1.57 ± 0.12 **gh**	5.03 ± 0.06 **de**	7.47 ± 0.15 **bc**
	WA + BS 100 ppm	1.13 ± 0.06 **gh**	3.60 ± 0.26 **f**	7.47 ± 0.06 **bc**
	WA + BS 1,000 ppm	1.83 ± 0.15 **g**	5.30 ± 0.26 **d**	7.93 ± 0.21 **b**
	WA + BS 10,000 ppm	0.97 ± 0.15 **h**	2.97 ± 0.81 **f**	4.37 ± 1.50 **e**
	PDA	3.13 ± 0.21 **f**	7.30 ± 0.60 **bc**	9.00 ± 0.00 **a**
**BS2**	WA	2.43 ± 0.12 **l**	4.23 ± 0.25 **h**	7.00 ± 0.00 **d**
	WA + BS 10 ppm	2.83 ± 0.29 **k**	4.90 ± 0.10 **g**	7.50 ± 0.00 **c**
	WA + BS 100 ppm	3.17 ± 0.15 **j**	5.00 ± 0.00 **g**	8.00 ± 0.00 **b**
	WA + BS 1,000 ppm	3.67 ± 0.29 **i**	5.83 ± 0.29 **f**	7.83 ± 0.29 **b**
	WA + BS 10,000 ppm	3.53 ± 0.06 **i**	6.33 ± 0.29 **e**	9.00 ± 0.00 **a**
	PDA	4.03 ± 0.06 **h**	7.03 ± 0.06 **d**	9.00 ± 0.00 **a**
**BS3**	WA	1.17 ± 0.06 **d**	4.03 ± 0.15 **c**	6.73 ± 0.06 **b**
	WA + BS 10 ppm	1.03 ± 0.06 **d**	4.13 ± 0.23 **c**	8.53 ± 0.15 **a**
	WA + BS 100 ppm	1.13 ± 0.06 **d**	4.53 ± 0.06 **c**	8.80 ± 0.10 **a**
	WA + BS 1,000 ppm	0.90 ± 0.10 **d**	4.30 ± 0.10 **c**	8.67 ± 0.12 **a**
	WA + BS 10,000 ppm	1.33 ± 0.15 **d**	5.27 ± 0.25 **b**	8.40 ± 0.10 **a**

PDA	1.20 ± 0.00 **d**	4.43 ± 0.21 **c**	8.90 ± 0.10 **a**

Values represent the average radial growth (cm) of three replicates ± standard deviation for each treatment. A two-way repeated measures ANOVA with Student-Newman-Keuls (SNK) test was performed for data comparison. Different letters indicate significant differences between treatments ($p<0.05$).

Table S8. Analysis of variance for the radial growth of *T. harzianum* TH01 in presence of different bioactive compounds (BS1, BS2, and BS3) and in the course of time (T with T1:24 h, T2:48 h, and T3:72 h).

Bioactive compounds	Treatment	Df	Sum Sq	Mean Sq	F value	Pr (>F)
BS1	Time of measurement (T)	2	10.843	5.421	44.090	1.16e-07 ***
	Biostimulants (B)	5	2.792	0.558	4.541	0.00746 **
	T *x* B	10	1.373	0.137	1.117	0.40155
	Residuals	18	2.213	0.123		
BS2	Time of measurement (T)	2	6.577	3.288	167.52	2.33e-12 ***
	Biostimulants (B)	5	1.169	0.234	11.91	3.40e-05 ***
	T *x* B	10	0.506	0.051	2.58	0.0385 *
	Residuals	18	0.353	0.020		
BS3	Time of measurement (T)	2	11.203	5.601	57.125	1.6e-08 ***
	Biostimulants (B)	5	2.610	0.522	5.324	0.00355 **
	T *x* B	10	4.853	0.485	4.949	0.00164 **
	Residuals	18	1.765	0.098		

*, **, ***, significant at $p < 0.05$, 0.01, and 0.001, respectively. The absence of asterisks indicates non-significant effects.

REFERENCES:

1. Abd El-Fattah, D. A.; Eweda, W. E.; Zayed, M. S.; Hassanein, M. K. Effect of Carrier Materials, Sterilization Method, and Storage Temperature on Survival and Biological Activities of Azotobacter Chroococcum Inoculant. *Ann. Agric. Sci.* **2013**, *58(2)*, 111-118. https://doi.org/10.1016/j.aoas.2013.07.001.
2. Singh, D. P.; Singh, H. B.; Prabha, R. *Microbial Inoculants in Sustainable Agricultural Productivity: Vol. 2: Functional Applications*; Springer: India; **2016.** https://doi.org/10.1007/978-81-322-2644-4.
3. Liffourrena, A. S.; Lucchesi, G. I. Alginate-Perlite Encapsulated Pseudomonas Putida A (ATCC 12633) Cells: Preparation, Characterization and Potential Use as Plant Inoculants. *J. Biotechnol.* **2018**, *278*, 28-33. https://doi.org/10.1016/j.jbiotec.2018.04.019.
4. Chen, B.; Yuan, M.; Qian, L. Enhanced Bioremediation of PAH-Contaminated Soil by Immobilized Bacteria with Plant Residue and Biochar as Carriers. *J. Soils Sediments* **2012**, *12*, 1350-1359. https://doi.org/10.1007/s11368-012-0554-5.
5. Quilliam, R. S.; Glanville, H. C.; Wade, S. C.; Jones, D. L. Life in the "charosphere" - Does Biochar in Agricultural Soil Provide a Significant Habitat for Microorganisms? *Soil Biol. Biochem.* **2013**, *65*, 287-293. https://doi.org/10.1016/j.soilbio.2013.06.004.
6. Glodowska, M. *Biochar as a Potential Inoculant Carrier for Plant-Beneficial Bacteria*; LAP Lambert Academic Publishing, **2005**. ISBN: 978-3-659-32047-7.
7. Sun, D.; Meng, J.; Liang, H.; Yang, E.; Huang, Y.; Chen, W.; Jiang, L.; Lan, Y.; Zhang, W.; Gao, J. Effect of Volatile Organic Compounds Absorbed to Fresh Biochar on Survival of Bacillus Mucilaginosus and Structure of Soil Microbial Communities. *J. Soils Sediments* **2015**, *15*, 271-281. https://doi.org/10.1007/s11368-014-0996-z.
8. Zhou, H.; Zhang, D.; Wang, P.; Liu, X.; Cheng, K.; Li, L.; Zheng, J.; Zhang, X.; Zheng, J.; Crowley, D.; van Zwieten, L.; Pan, G. Changes in Microbial Biomass and the Metabolic Quotient with Biochar Addition to Agricultural Soils: A Meta-Analysis. *Agric. Ecosyst. Environ.* **2017**, 239, 80-89.

https://doi.org/10.1016/j.agee.2017.01.006.

9. Hill, R. A.; Hunt, J.; Sanders, E.; Tran, M.; Burk, G. A.; Mlsna, T. E.; Fitzkee, N. C. Effect of Biochar on Microbial Growth: A Metabolomics and Bacteriological Investigation in E. Coli. *Environ. Sci. Technol.* **2019**, *239*, 80-89. https://doi.org/10.1021/acs.est.8b05024.
10. Kashyap, P. L.; Xiang, X.; Heiden, P. Chitosan Nanoparticle Based Delivery Systems for Sustainable Agriculture. *Int. J. Biol. Macromol*. **2015**, *77*, 36-51. https://doi.org/10.1016/j.ijbiomac.2015.02.039.
11. Kumaraswamy, R. V.; Kumari, S.; Choudhary, R. C.; Pal, A.; Raliya, R.; Biswas, P.; Saharan, V. Engineered Chitosan Based Nanomaterials: Bioactivities, Mechanisms and Perspectives in Plant Protection and Growth. *Int. J. Biol. Macromol*. **2018,** *113*, 494-506. https://doi.org/10.1016/j.ijbiomac.2018.02.130.
12. Perez, J. J.; Francois, N. J.; Maroniche, G. A.; Borrajo, M. P.; Pereyra, M. A.; Creus, C. M. A Novel, Green, Low-Cost Chitosan-Starch Hydrogel as Potential Delivery System for Plant Growth-Promoting Bacteria. *Carbohydr. Polym.* **2018**, *202,* 409-417. https://doi.org/10.1016/j.carbpol.2018.07.084.
13. Nuti, M.; Giovannetti, G. Borderline Products between Bio-Fertilizers/Bio-Effectors and Plant Protectants: The Role of Microbial Consortia. *J Agric Sci Technol A* **2015**, *5*, 305–315. https://doi.org/10.17265/2161-6256/2015.05.001.
14. Duo, L. A.; Liu, C. X.; Zhao, S. L. Alleviation of Drought Stress in Turfgrass by the Combined Application of Nano-Compost and Microbes from Compost. *Russ. J. Plant Physiol.* **2018**, *65*, 419-426. https://doi.org/10.1134/S102144371803010X.
15. Jampílek, J.; Králová, K. Nanomaterials for Delivery of Nutrients and Growth-Promoting Compounds to Plants. In *Nanotechnology: An Agricultural Paradigm*; Springer: Singapore; **2017**. https://doi.org/10.1007/978-981-10-4573-8_9.
16. Prasad, R.; Bhattacharyya, A.; Nguyen, Q. D. Nanotechnology in Sustainable Agriculture: Recent Developments, Challenges, and Perspectives. *Front. Microbiol.* **2017**, *8,* 1014. https://doi.org/10.3389/fmicb.2017.01014.
17. Daza, A.; Santamaría, C.; Rodríguez-Navarro, D. N.; Camacho, M.; Orive, R.; Temprano, F. Perlite as a Carrier for Bacterial Inoculants. *Soil Biol. Biochem.* **2000**, 32(4), 567-572. https://doi.org/10.1016/S0038-0717(99)00185-6.
18. Nehra, V.; Choudhary, M. A Review on Plant Growth Promoting Rhizobacteria Acting as Bioinoculants and Their Biological Approach towards the Production of Sustainable Agriculture. *J. Appl. Nat. Sci.* **2015**, *7(1),* 540-556. https://doi.org/10.31018/jans.v7i1.642.
19. Backer, R.; Rokem, J. S.; Ilangumaran, G.; Lamont, J.; Praslickova, D.; Ricci, E.; Subramanian, S.; Smith, D. L. Plant Growth-Promoting Rhizobacteria: Context, Mechanisms of Action, and Roadmap to Commercialization of Biostimulants for Sustainable Agriculture. *Front. Plant Sci.* **2018**, 9, 1473. https://doi.org/10.3389/fpls.2018.01473.
20. Ding, Y.; Liu, Y.; Liu, S.; Li, Z.; Tan, X.; Huang, X.; Zeng, G.; Zhou, L.; Zheng, B. Biochar to Improve Soil Fertility. A Review. *Agron. Sustain. Dev.* **2016**, *36*, 36. https://doi.org/10.1007/s13593-016-0372-z.
21. Latini, A.; Bacci, G.; Teodoro, M.; Gattia, D. M.; Bevivino, A.; Trakal, L. The Impact of Soil-Applied Biochars From Different Vegetal Feedstocks on Durum Wheat Plant Performance and Rhizospheric Bacterial Microbiota in Low Metal-Contaminated Soil. *Front. Microbiol.* **2019**, *10,* 2694. https://doi.org/10.3389/fmicb.2019.02694.
22. Lehmann, J.; Rillig, M. C.; Thies, J.; Masiello, C. A.; Hockaday, W. C.; Crowley, D. Biochar Effects on Soil Biota - A Review. *Soil Biol. Biochem.* **2011**, *43(9)*, 1812-1836. https://doi.org/10.1016/j.soilbio.2011.04.022.
23. Egamberdieva, D.; Wirth, S.; Behrendt, U.; Abd-Allah, E. F.; Berg, G. Biochar Treatment Resulted in a Combined Effect on Soybean Growth Promotion and a Shift in Plant Growth Promoting Rhizobacteria. *Front.*

Microbiol. **2016**, *7*, 209. https://doi.org/10.3389/fmicb.2016.00209.

24. Muter, O.; Grantina-Ievina, L; Makarenkova, G.; Vecstaudza, D.; Strikauska, S.; Selga, T.; Ksparinskis, R; Stelmahere, S.; Steiner, C. Effect of Biochar and Trichoderma Application on Fungal Diversity and Growth of Zea Mays in a Sandy Loam Soil. *Environ. Exp. Biol.* **2017**, *15*: 289-296. https://doi.org/10.22364/eeb.15.30.
25. Ribera, J.; Gandía, M.; Marcos, J. F.; Bas, M. D. C.; Fink, S.; Schwarze, F. W. M. R. Effect of Trichoderma-Enriched Organic Charcoal in the Integrated Wood Protection Strategy. *PLoS One* **2017**, *12(8)*, e0183004. https://doi.org/10.1371/journal.pone.0183004.
26. Malusá, E.; Sas-Paszt, L.; Ciesielska, J. Technologies for Beneficial Microorganisms Inocula Used as Biofertilizers. *Scientific World Journal*. **2012**, *2012*: 491206. https://doi.org/10.1100/2012/491206.
