# Supplementary figures and images for: Identification of Beneficial Microbial Consortia and Bioactive Compounds with Potential as Plant Biostimulants for a Sustainable Agriculture
Source: Microorganisms. 2021 Feb 19;9(2):426. doi: 10.3390/microorganisms9020426 (PMC7922931; doi:10.3390/microorganisms9020426)

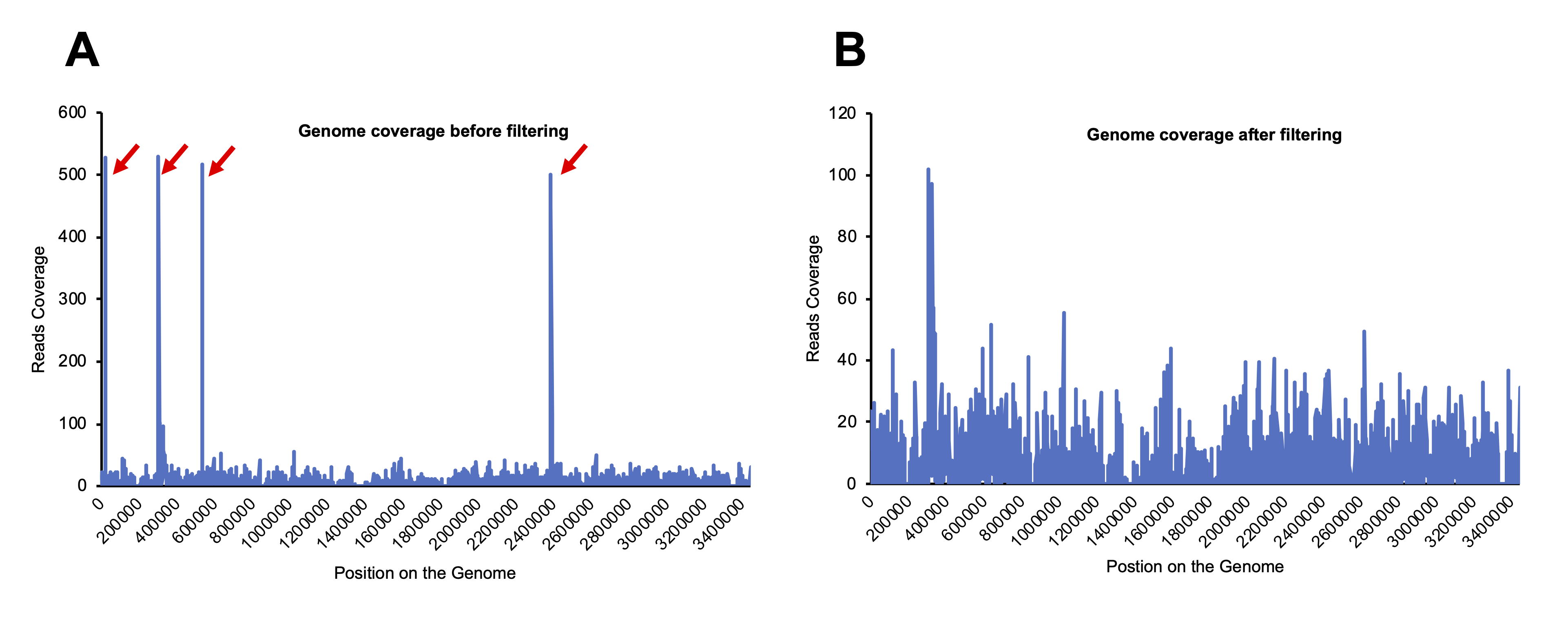

Supplement: Supplementary file 1 [file microorganisms-09-00426-s001.zip › Supplementary files /Figure S1.tiff]
